# Investigating a new *Dillenia ovata* endophytic bacteria to produce antioxidants and anti-diabetes activity *in vitro* and *in vivo*

**DOI:** 10.1016/j.btre.2025.e00921

**Published:** 2025-08-20

**Authors:** Linh Chi Tran, Chong Kim Thien Duc, Tuan Trong Nguyen, Duy Toan Pham, Danh Thai Luu, Trang Thi Xuan Dai

**Affiliations:** a Institute of Food and Biotechnology, Can Tho University, Can Tho City 94000, Vietnam; b Faculty of Medicine, Nam Can Tho University, Can Tho City 94000, Vietnam; c College of Natural Sciences, Can Tho University, Can Tho City 94000, Vietnam; d College of Agriculture, Can Tho University, Can Tho City 94000, Vietnam

**Keywords:** Antioxidants, Anti-diabetes, *Bacillus* sp., Box-Behnken, *Dillenia ovata*, Endophytic bacteria

## Abstract

A novel endophytic bacterium, *Bacillus* sp. DO-R5, with strong antioxidant and anti-diabetic properties, was isolated from *Dillenia ovata*, a traditional Vietnamese medicinal plant. *Bacillus* sp. DO-R5 was identified using morphological characteristics, SEM, and *16S rRNA* sequencing, demonstrated the highest bioactivity among the 19 isolates. Optimal fermentation conditions (8.9 g/L D-glucose, pH 7.5, 38 °C, and 70.5 h) were determined using the Box-Behnken design. Under these conditions, *Bacillus* sp. DO-R5 significantly enhanced the production of bioactive compounds including diosmin, rutin, and chlorogenic acid, and exhibited improved *in vitro* antioxidant, anti-inflammatory, and anti-diabetic activities. *In vivo*, the extract effectively reduced blood glucose, regulated lipid profiles, supported weight control, and protected organs. These findings emphasize the potential therapeutic use of *D. ovata* endophytic bacteria as a natural source of anti-diabetic drugs.

## Highlights

• *Dillenia ovata* is a plant species being conserved in An Giang, Vietnam.
• Endophytic bacteria in *Dillenia ovata* offered up new avenues for natural medicine.
• Optimal conditions for *Bacillus* sp. DO-R5 to create antioxidants have been identified.
• *Bacillus* sp. DO-R5 generates various bioactive flavonoid compounds.
• *Bacillus* sp. DO-R5 extract could reduce diabetes and its complications in mice.

## Graphical Abstract

**Figure:**
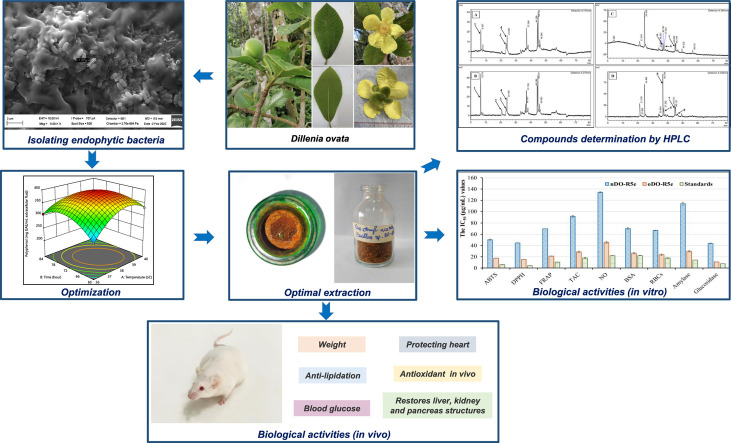

## 1 Introduction

Hyperglycemia is a characteristic of diabetes mellitus, a chronic disease, leading to lipid, carbohydrate, and protein metabolic abnormalities caused by insulin insensitivity. Diabetes can cause a variety of serious consequences relating to neurological, renal, liver, retinal, and cardiovascular complications [1]. Chronic hyperglycemia induces oxidative stress, which causes problems and organ damage [2]. Furthermore, diabetic people are more susceptible to elevated cholesterol and triglycerides that cause dyslipidemia [3]. Up to date, anti-diabetic therapies include lowering oxidative stress, inflammation, and delaying carbohydrate metabolism. Natural compounds, commonly isolated from medicinal plants, have been recommended to minimize oxidative stress, inflammation, and carbohydrate metabolism enzyme such as α-amylase and α-glucosidase [4,5].

*Dillenia ovata* Wall.ex Hook.f. et Thomson (DO) is a perennial woody plant that thrives in hilly regions and is found in Southeast Asian countries. For many years, the plant has been utilized in traditional treatments to treat diarrhea, dysentery, infections, arthritis, diabetes, and hepatitis. Phytochemical composition studies have revealed that DO contains alkaloids, tannins, flavonoids, glycosides, and phenols [6]. Accordingly, DO inhibited α-amylase and α-glucosidase, and was found to include antioxidants, anti-acetylcholinesterase, and phenolics with drug-like properties [7]. However, medicinal plants, particularly DO*,* frequently have a long growth season, which leads to depletion and unsecured sustainable raw material sources. This inspires scientists to study and discover new sources of therapeutic compounds, with endophytic bacteria in medicinal plants being a promising candidates.

Endophytic bacteria are microorganisms that reside in plant tissues during any stage of the host plant’s cycle and do not cause damage to the host plant [8]. It has been reported that flavonoids, alkaloids, isoprenoids, and indoles have antioxidant, antimicrobial, and lipid peroxidation properties [[9], [10], [11]]. The positive relationship between bacterial endophytes and 40 families with 86 plants was identified, including *Bacillus, Pantoea*, and *Pseudomonas, which* were the most prevalent genera, accounting for 58.92 percent [12]. *Bacillus, Pseudomonas*, and *Paenibacillus* are microorganisms that can affect plant metabolism, development, and stress tolerance [13]. The antibacterial, antifungal, and anticancer activity of the endophytic bacteria have been extensively explored [[14], [15], [16], [17], [18], [19], [20], [21], [22]]. Research on endophytic bacteria capable of blocking key carbohydrate metabolism enzymes or reducing blood glucose levels have also been studied [[23], [24], [25]]. Thus, baterial endophytes have been recognized as natural source of beneficial compounds.

In this study, the endophytic bacterial strain DO-R5 with the highest total polyphenol and flavonoid contents were isolated from DO root tissue and identified as *Bacillus* sp. DO-R5 using morphology and molecular biology. The *Bacillus* sp. DO-R5 was then employed to optimize antioxidant sources under experimental culture conditions using response surface methodology (RSM) model. Finally, the anti-diabetic properties of *Bacillus* sp. DO-R5 ethyl acetate extract from cell-free supernatant were investigated both *in vitro* and *in vivo*.

## 2 Materials and methods

### 2.1 Plant samples collecting and identifyting

**Fig. 1:**
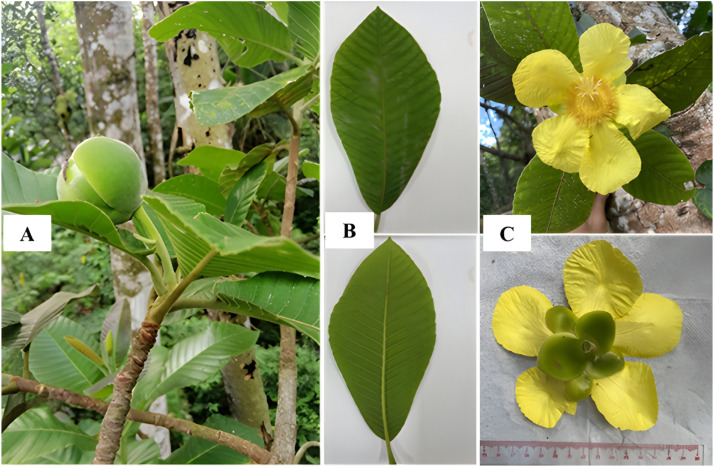
Morphology of *Dillenia ovata* A, habit; B, leaves, C, flowers.

*Dillenia ovata* Wall.ex Hook.f. et Thomson (DO) plant was collected in July 2024 from An Giang province, Vietnam. The plant species was regconized using botanical criteria and a specimen was kept (sample label: AG_DO202207100001) at the Laboratory of Medicinal Plants and Bioassays, Department of Biology, College of Natural Sciences, Can Tho University. DO roots, stems, and leaves (Fig. 1) were cut and kept in a plastic bag. The samples were directly transported to the lab in order to isolate bacterial endophytes.

### 2.2 Bacterial endophytes isolation

Plant samples were first washed thoroughly under running tap water, rinsed with sterile distilled water, and cut into small segments of 3–4 cm. The segments were surface-sterilized with 70 % ethanol for 30 s, rinsed 3–4 times with sterile distilled water, and treated with 3 % hydrogen peroxide for 3 min. Finally, the samples were rinsed 4–5 times with sterile distilled water to remove residual chemicals. To verify the efficiency of surface sterilization, 100 µL of the final rinse water was spread onto potato dextrose agar (PDA) plates and incubated at 30 °C for 48 h. The absence of microbial growth confirmed successful sterilization.

After sterilization, plant tissues were ground in a sterile mortar and pestle. Sterile distilled water (2–3 mL) was added, mixed thoroughly, and allowed to stand for 10–12 min to settle debris. An aliquot of 100 µL of the supernatant was inoculated into test tubes containing 10 mL of semi-solid PDA medium (0.15 % agar), and the tubes were incubated at 30 °C for 48 h. The appearance of a thin whitish pellicle 2–4 mm below the surface of the medium indicated the growth of endophytic bacteria. Pure cultures were obtained by repeated streaking, and colony morphology (shape, size, color, margin, elevation) as well as cellular characteristics (shape, size, Gram reaction) were examined.

### 2.3 Total polyphenol and flavonoid content

#### 2.3.1 Sample preparation

Pure cultures of endophytic bacterial strains were cultivated in potato dextrose broth (PDB, pH 7.0) at 30 °C for 24 h. Seed cultures were adjusted to an optical density of 0.5 at 600 nm (OD_600_ = 0.5). Each strain (200 µL) was inoculated into 9.8 mL of fresh PDB medium (pH 7.0) and incubated at 30 °C for 24 h with shaking at 200 rpm. Following incubation, cultures were centrifuged at 3000 rpm for 10 min to remove bacterial cells, and the resulting cell-free supernatants were collected. These supernatants were stored in sterile glass bottles at 4 °C until further analyses.

#### 2.3.2 Determination of total polyphenol and flavonoid content

The total polyphenol content (TPC) and flavonoid content (TFC) were evaluated according to the previous methods [26,27]. TPC and TFC were expressed as milligrams of gallic acid equivalents per milliliter of cell-free supernatant (mg GAE/mL cell-free supernatant) and quercetin equivalents per milliliter of cell-free supernatant (mg QE/mL cell-free supernatant).

### 2.4 Identification of the endophytic bacterial strain

#### 2.4.1 Morphological identification

Morphological characteristics of the bacterial cells were observed using light microscopy (Olympus, Japan) and scanning electron microscopy (SEM; Carl Zeiss, Germany). Gram staining was performed sequentially with crystal violet, iodine solution, decolorization with alcohol, and counterstaining with safranin, followed by rinsing with distilled water. Gram-positive bacteria appeared violet-blue, while Gram-negative bacteria appeared pink.

#### 2.4.2 DNA identification

Endophytic bacterial strains from DO exhibiting the highest *in vitro* antioxidant activity (polyphenol and flavonoid production) were selected for molecular identification. Cell morphology was examined using light microscopy (CX21FS1, Olympus, Japan) and scanning electron microscopy (Carl Zeiss, Germany). Genomic DNA was extracted, and the *16S rRNA* gene was amplified using the universal primers 27F (5′-AGAGTTTGATCCTGGCTCAG-3′) and 1492R (5′-TACGGTTACCTTGTTACGACTT-3′). This primer pair amplifies nearly the full-length *16S rRNA* gene (∼1500 bp), covering most variable regions (V1–V9), which provide high-resolution taxonomic information for bacterial identification at the genus and, in some cases, species level [28]. PCR was performed under the following conditions: initial denaturation at 95 °C for 5 min; 35 cycles of 94 °C for 1 min, 53 °C for 1 min, and 72 °C for 1.5 min; followed by a final extension at 72 °C for 5 min [27]. Sequencing was carried out at T and N Biosolution Company Limited (Ho Chi Minh City, Vietnam).

The obtained sequences were compared with the *16S rRNA* gene database in GenBank using BLASTN to determine similarity with reference strains. *Sphingobium lactosutens* DS20 was used as the outgroup. Multiple sequence alignment was performed with BioEdit v7.2.1, yielding 1305 aligned nucleotides. Phylogenetic trees were reconstructed using the Maximum Likelihood method with the Hasegawa–Kishino–Yano model, and bootstrap support was calculated with 1000 replications. Final phylogenetic analysis was conducted with MEGA v11.0.13, providing robust taxonomic placement of the endophytic bacterial strains.

### 2.5 Optimization of the culture conditions of *Bacillus* sp. DO-R5 using One factor at a time approach

The preliminary studies aimed to determine the culture parameters (temperature, time, glucose, and initial pH) that would have significant effect on the culture to produce high TPC and TFC. Based on prior fermentation conditions [27], the variables were temperature (from 30 to 42 °C), time (from 12 to 84 h), glucose content (from 0 to 35 g/L), and initial pH (from 5 to 8).

### 2.6 Experimental design

A four-factor, three-level of RSM (Box–Behnken design; BBD) was applied to determine the ideal conditions for *Bacillus* sp. DO-R5 fermentation. The optimal conditions with four variables were designed as X_1_ (temperature), X_2_ (time), X_3_ (glucose concentration), X_4_ (initial pH). TPC and TFC, two predicted responses, were assigned the designations Y_1_ and Y_2_, respectively. Each factor with three levels: low (−1), medium (0), and high (1) was evaluated. The relationship between the independent variables and TPC or TFC production was modeled using a second-order polynomial equation, as shown in Eq. (1)

(1) $$Y_{1}\;or\;Y_{2}=\beta_{0}+\sum_{i=1}^{4}\beta_{i0}.X_{i}+\sum_{i=1;j=2,i<j}^{4}\beta_{ij}X_{i}.X_{j}+\sum_{i=1}^{4}\beta_{ii}.X_{i}^{2}$$

In this equation, Y_1_ (TPC) or Y_2_ (TFC) indicates the response value corresponding to each combination of factor levels. The coefficients β_0_, β_i_, and β_j_ represent the regression terms, and X_1_, X_2_, X_3_, and X_4_ are the experimental factors.

### 2.7 Crude extraction of cell-free supernatant of *Bacillus* sp. DO-R5

*Bacillus* sp. DO-R5 was culture under optimal conditions obtained in Section 2.6 and normal culture condition (30 °C, 24 h, 20 g/L glucose, pH 7). The cell-free supernatant was prepared using the same steps described above. Ethyl acetate was mixed with the cell-free supernatant with a ratio of 1:1 (v/v). The liquid phase containing the extract was separated with a funnel. A rotary evaporator set to 90 rpm and 50 °C was used to evaporate the extract. The extract of cell-free supernatant of *Bacillus* sp. DO-R5 under optimal growth conditions symbolizes oDO-R5e, while under normal culture conditions stands for nDO-R5e, were subsequently stored for later research at 4 °C [27].

### 2.8 Compound determination of DO-R5 extracts by HPLC

***The standard calibration curves.*** 1 mg of each reference (hesperidin, rutin, diosmin, quercetin, kaempferol, gallic acid, vanillic acid, caffeic acid, and chlorogenic acid) was dissolved in MeOH to final concentration of 1 mg/mL. Five concentrations (concentration range from 0.0156 to 0.25 mg/mL for hesperidin, rutin, diosmin, kaempferol, vanillic acid, caffeic acid, and chlorogenic acid; concentration range from 0.00156 to 0.025 mg/mL for quercetin, and gallic acid) were used to create standard calibration curves. Every sample was run three times.

***HPLC analysis.*** The Shimadzu Prominence-i LC-2030 HPLC system (Shimadzu, Tokyo, Japan) was used for the analysis. The system was controlled using LabSolution software. The sample solution in MeOH (2 mg extract/mL) were injected into Phenomenex (Torrance, California, USA) C18 column (5 µm, 4.6 × 250 mm), attached to a pre-column (C18, 4 × 10 mm) at 30 °C. The samples were measured at two wavelengths of 270 and 340 nm. The mobile phase consisted of 2 % acetic acid (A) and methanol (B) and the composition gradient was: 10 % B for up to 10 min and varied to obtain 20, 30, 50, 60, 70, 20 % and 10 % B at 20, 30, 40, 50, 60, 70 and 80 min respectively. The injection volume is 5 μL with a flow rate of 1.0 mL/min.

The identification of the individual components of the extract was achieved by comparing the retention times with the standards and the calibration curve was used to quantify the components (Table 1). The results were expressed as µg/mg extract, each extract was analyzed three times.

**Table 1:** Chromatographic parameters of the method used for the quantification of compounds in the extract.

Standard substance	RT	Concentration range (mg/mL)	Standard curve equation	Correlation coefficient (R^2^)
Gallic acid	6.295	0.00156–0.025	y = 17609400x – 8046.63	0.9938556
Chlorogenic acid	20.779	0.0156–0.25	y = 6549130x – 578999.5	0.9992826
Vanillic acid	23.589	0.0156–0.25	y = 8459080x – 35947.6	0.9995220
Caffeic acid	24.212	0.0156–0.25	y = 10389900x – 50209.6	0.9996896
Hesperidin	35.912	0.0156–0.25	y = 1408210x + 1264.63	0.9999230
Rutin	36.296	0.0156–0.25	y = 7499020x – 5024.32	0.9998492
Diosmin	37.739	0.0156–0.25	y = 299216x – 369.405	0.9998598
Quercetin	44.274	0.00156–0.025	y = 10788100x + 737.967	0.9995183
Kaempferol	48.340	0.0156–0.25	y = 14161800x - 107480	0.9990783

### 2.9 *In vitro* assays for antioxidant, anti-inflammatory, α-amylase and α-glucosidase inhibitory activities of *Bacillus* sp. DO-R5 extract

The antioxidant properties of both the nDO-R5e and oDO-R5e extracts were evaluated using 2,2’-azino-bis-3-ethylbenzthiazoline-6-sulfonic acid (ABTS^•+^), 2,2-diphenyl-1-picrylhydrazyl (DPPH), ferric reducing-antioxidant power (FRAP), total antioxidant capacity (TAC), and nitric oxide (NO^•^) scavenging ability, according to standard methods described by Anh et al. (2021) [29]. Ascorbic acid was used as the positive control. The antioxidant activities of the extract were compared to those of ascorbic acid by calculating the concentration (μg/mL) by which the extract or ascorbic acid can reduce or neutralize 50 % of free radicals (IC_50_, inhibitory concentration of 50 %).

The anti-inflammatory activity of both the nDO-R5e and oDO-R5e extracts was based on the inhibition of protein denaturation and erythrocyte protection based on the methods of Anyasor et al. (2019) [30] and Aidoo et al. (2021) [31]. The anti-inflammatory activity of extract was expressed as concentration of extract or standard (diclofenac) which could inhibit 50 % of protein denaturation or erythrocyte protection (IC_50_) [31].

The α-amylase and α-glucosidase activities were assayed following Deveci et al. (2021) [32]. Positive controls for both assays were acarbose. The inhibitory concentration of 50 % (IC_50_) was expressed as the activity of α-amylase or α-glucosidase.

### 2.10 *In vivo* anti-diabetic effect of DO-R5 extracts

#### 2.10.1 Induction of diabetic mice

Eight-week-old male *Mus musculus* var. Albino laboratory mice, weighing approximately 30 to 35 g, were provided by the Pasteur Institute of Ho Chi Minh City and maintained in the laboratory with glass cages measuring 25 × 20 × 15 cm at room temperature, with a 12/12 h light/dark cycle. The mice were fed conventional food pellets and water during the experiment, with the exception of the fasting period. Animal care and handling procedures were established for the use of laboratory animals (National Research Council (US) Committee for the Update of the Guide for the Care and Use of Laboratory Animals 2011) [33] and the research protocol was approved by the Animal Ethics Committee of Can Tho University (CTU-AEC), Can Tho University, Can Tho City, Vietnam (Approval code: CTU-AEC24017).

Before fasting for 18 h, mice were intraperitoneally injected with 135 mg/kg body weight (BW) of alloxan monohydrate (AM) for 3 days. After 7 days since the last injection, mice blood was collected and measured for blood glucose level by using an ACCU-CHEK® Active glucometer (Roche Diagnostics, Switzerland). Mice with glucose levels of 200-400 mg/dL were designated diabetic mice for the following experiment [34].

Forty-two male mice were randomly assigned to six groups (n=7) and then treated as follows. Group I: normal mice, oral administration of distilled water (N). Group II: AM-induced diabetic mice, oral administration of distilled water (D). Group III: AM-induced diabetic mice orally treated with glibenclamide at the dose of 10 mg/kg BW (D, Gli). Group IV, V, and VI: AM-induced diabetic mice were orally administered with 100 mg (D, oDO-R5e, 100), 200 mg (D, oDO-R5e, 200), and 400 mg (D, oDO-R5e, 400) oDO-R5e/kg BW daily, respectively. Animals were administered with glibenclamide or oDO-R5e twice daily for 21 days.

#### 2.10.2 Biochemical analysis and histopathological examination

After 21 days of treatment period, mice were sacrificed using diethyl ether after 24 h of fasting, blood and organs were collected for further analysis and microscopic observation. Plasma was isolated from the blood sample by centrifugation for 10 min at 3000 rpm at room temperature. Glucose levels were determined by ACCU-CHEK® Active. The semi-automated machine TOYOBO TYB-18 (Furuno Electric Co., Ltd, Japan) was used to monitor the plasma lipid profile, which included total cholesterol (TC), triglycerides (TG), high-density lipoprotein cholesterol (HDL-C), alanine transaminase (ALT), and aspartate transaminase (AST). LDL-C (low-density lipoprotein cholesterol) and VLDL-C (very-low-density lipoprotein cholesterol) were calculated using the formulas: LDL-C = [(TC-HDL cholesterol)-TG/5] and VLDL-C = TG/5 [51].

Furthermore, atherogenic index of plasma (AIP), atherogenic coefficient (AC), cardiac risk ratio (CRR), and cardioprotective index (CPI) were determined by the following formula AIP = Log(TG/HDL cholesterol), AC = (TC – HDL cholesterol)/HDL cholesterol, CRR = TC/HDL cholesterol, and CPI = HDL cholesterol/LDL cholesterol [35,36].

The markers of oxidative stress, malondialdehyde (MDA) [37] and glutathione (GSH) [38], were analysis in the liver, kidneys, and pancreas of mice. The effects of oDO-R5e on MDA and GSH production were represented as nM/g tissue of MDA, and GSH was computed using a linear regression equation with MDA and GSH standards, respectively.

A small sample of the liver, kidneys, and pancreas was obtained, and histopathological changes were identified under microscope after staining with hematoxylin and eosin using the previous approach [39].

### 2.11 Statistical analysis

Each experiment included at least three replicates. Means and standard deviations were used to describe the experimental data. The experimental data was analyzed using the Minitab 16 program (ANOVA-Tukey's).

A significant difference was considered with a *p*-value ≤ 0.05. Graphs were created with Microsoft Excel 2016. BBD and the RSM experimental results were analyzed by Design-Expert Software version 11 from Alfasoft AB in Göteborg, Sweden.

## 3 Results

### 3.1 Isolation of endophytic bacteria

The 19 bacterial strains were isolated from DO roots (9 strains), stems (4 strains), and leaves (6 strains). Surface sterilization was carried out on the selected tissue to eliminate surface microbial flora such as epiphytes, and all of them revealed endophytes. The bacterial strains were coded DO-Rx, DO-Sx, and DO-Lx (DO: *D. ovata*, R: root, S: stem, L: leaf, x: The number of the bacterial strains).

The phenotypic characteristics of the DO endophytic bacteria strains are shown in Table 2. The 18 (94.74 %) strains developed circular colonies, while one (5.26 %) had irregular colonies. The colony colors were milk-white (4 strains), ivory (6 strains), off white (5 strains), dark yellow (2 strains), and light yellow (2 strains), with relative ratios of 21.05 %, 31.57 %, 26.32 %, 10.53 %, and 10.53 %. Nine (47.37 %) endophytic bacterial strains had flat elevation, whereas ten (52.63 %) had convex elevation. The 18 (94.74 %) strains with colonies were entire, but one (5.26 %) strain with an undulate margin. All bacterial strains exhibited rod cell morphologies. Among the 19 strains, there were 15 with negative Gram (78.95 %) and 4 with positive Gram (21.05 %).

**Table 2:** Phenotypic characteristics of the *D. ovata* endophytic bacteria strains.

Targets	Characteristics	Number of strains	Percentage (%)
Colony shape	Circular	18	94.74
	Irregular	1	5.26
Colony color	Milk-white	4	21.05
	Ivory	6	31.57
	Off white	5	26.32
	Dark yellow	2	10.53
	Light yellow	2	10.53
Elevation	Flat	9	47.37
	Convex	10	52.63
Margin	Entire	18	94.74
	Undulate	1	5.26
Cell shape	Rod-shaped	19	100
Gram reaction	Negative	15	78.95
	Positive	4	21.05

### 3.2 Screening *D. ovata* endophytic bacterial strains with high total polyphenol and flavonoid content

**Fig. 2:**
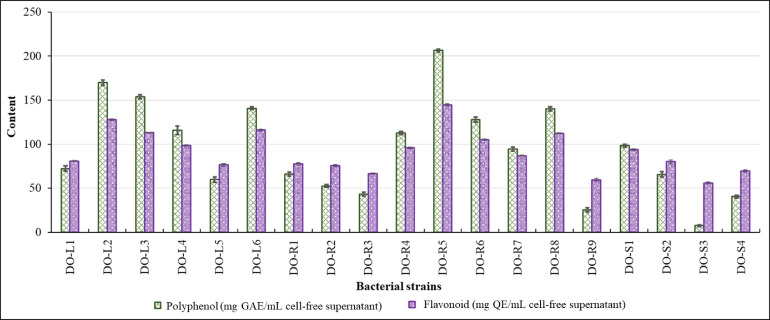
Polyphenols and flavonoids content are produced by *D. ovata* isolated endophytic bacterial strains.

Polyphenols and flavonoids, two crucial natural compounds, can help prevent and treat a variety of ailments, including cancer, gout, hepatitis, and diabetes [40]. All the 19 bacterial strains could produce TPC (7.51 to 206.52 mg GAE/mL) and TFC (58.65 to 144.81 mg QE/mL) (Fig. 2). Among them, the DO-R5, DO-L2, DO-L3 strains had the highest TPC and TFC production capabilities.

### 3.3 Identification of the endophytic bacteria

**Fig. 3:**
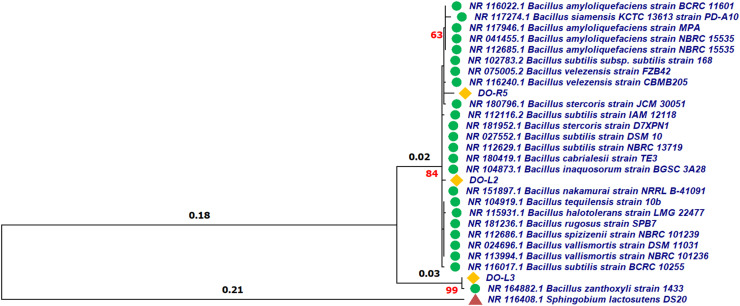
Phylogenetic tree of bacterial strains DO-R5, DO-L2 and DO-L3 based on *16S rRNA* gene sequences.

Bacterial strains DO-R5, DO-L2, and DO-L3 had the ability to produce polyphenols and flavonoids more effectively than the other bacterial strains, so they were selected for DNA extraction and sequencing of the *16S rRNA* gene. Based on the characteristics of colonies and cells of the bacteria (Table 2) and the gene information of *16S rRNA*, bacterial strains DO-R5, DO-L2, and DO-L3 were identified as *Bacillus* sp. (Fig. 3). Information on DO-R5, DO-L2, and DO-L3 bacterial strains from this study was published with accession numbers shown in Table 3.

**Table 3:** Information on bacterial strains DO-R5, DO-L2, and DO-L3 on the GenBank nucleotide database of NCBI.

Samples	GenBank	Link
*Bacillus* sp. DO-R5	PQ533190	https://www.ncbi.nlm.nih.gov/nuccore/PQ533190
*Bacillus* sp. DO-L2	PQ533464	https://www.ncbi.nlm.nih.gov/nuccore/PQ533464
*Bacillus* sp. DO-L3	PQ533189	https://www.ncbi.nlm.nih.gov/nuccore/PQ533189

**Fig. 4:**
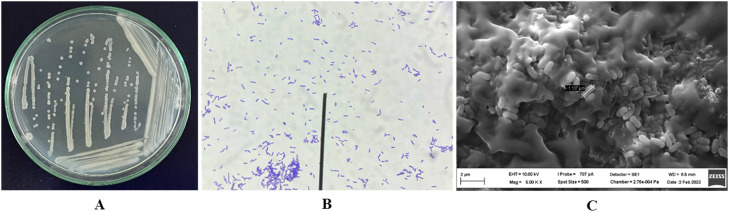
Morphological characteristics of DO-R5 bacterial strain.

Notes: (A) Colony morphology of bacteria in a 10 cm petri dish, (B) Cell morphology of bacteria with magnification of 400 times, (C) Size of bacteria observed by microscope scanning electron micrograph (SEM).

The bacterial strain DO-R5, produced the highest content of polyphenols and flavonoids, was selected the for further study. After 24 h of culture on PDA medium, the bacterial strain DO-R5 showed colonies with irregular shape, off white color, flat elevation, undulate margin, and colony size from 0.5 to 1.5 mm. DO-R5 was rod shape with cell size approximately 0.45 µm × 1.12 µm and Gram positive (Fig. 4).

### 3.4 Evaluation of variables affecting TPC and TFC production of *Bacillus* sp. DO-R5

**Fig. 5:**
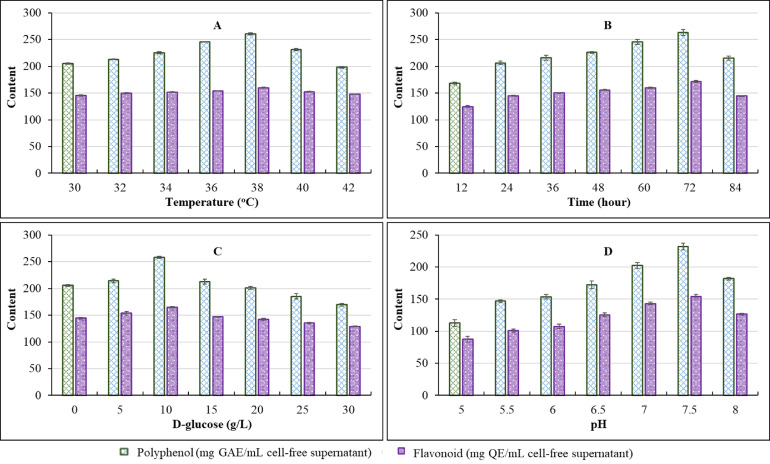
Polyphenol and flavonoid contents in the cell-free supernatant of DO-R5 under different culture conditions.

Note: (A) Temperature, (B) Time, (C) D-glucose and (D) pH on the ability to produce polyphenols and flavonoids, respectively.

The most significant effects on the TPC and TFC production of *Bacillus* sp. DO-R5 were determined by four experimental factors, including culture temperature, culture time, glucose concentration, and starting pH. Fig. 5 displays the influence of four factors on TPC and TFC production during optimization using the typical one-factor-at-a-time technique. The experimental findings reveal that when the temperature was 38 °C, the yield of TPC and TFC reached maximum, as illustrated in Fig. 5**A**. Fig. 5**B** shows that TPC and TFC production reached their peaks at 72 h of culture. The highest content of TPC and TFC values were obtained at 10 g/L glucose concentration, as shown in Fig. 5**C**. Fig. 5**D** shows that the maximum TPC and TFC yields occurred at pH 7.5.

### 3.5 Experimental design

PDB was used to assess the relative significance of four factors of culture as independent variables: X_1_ (temperature), X_2_ (time), X_3_ (glucose concentration), X_4_ (initial pH), with codes A, B, C, D, respectively. Table 4 shows the ranges and numbers of the independent variables in their code forms. Table 5 shows the results of 29 experimental runs with various combinations of the four components. The linear regression equation was calculated as follows:

$$Y_{\text{Polyphenol}}$$

$$=\;-49815.52\;+\;1448.92\;\times \;A\;+\;93.87\;\times \;B\;+\;195.31\;\times \;C\;+\;4880.50\;\times \;D\;-\;1.01\;\times \;A\;\times \;B\;-\;1.91\;\times \;A\;\times \;C\;-\;46.05\;\times \;A\;\times \;D\;+\;0.14\;\times \;B\;\times \;C\;-\;1.54\;\times \;B\;\times \;D\;-\;14.32\;\times \;C\;\times \;D\;-\;13.26\;\times \;A^{2}\;-\;0.32\;\times \;B^{2}\;-\;1.39\;\times \;C^{2}\;-\;192.35\;\times \;D^{2}$$

$$Y_{\text{Flavonoid}}$$

$$=\;-30667.70\;+\;892.46\;\times \;A\;+\;59.57\;\times \;B\;+\;115.18\;\times \;C\;+\;2989.86\;\times \;D\;-\;0.645313\;\times \;A\;\times \;B\;-\;1.14\;\times \;A\;\times \;C\;-\;29.64\;\times \;A\;\times \;D\;+\;0.11\;\times \;B\;\times \;C\;-\;0.97\;\times \;B\;\times \;D\;-\;8.71\;\times \;C\;\times \;D\;-\;8.01\;\times \;A^{2}\;-\;0.20\;\times \;B^{2}\;-\;0.79\;\times \;C^{2}\;-\;114.21\;\times \;D^{2}$$

**Table 4:** Factors and levels in response surface analysis.

Factors	-1	0	(+1)	ΔX_i_
Culture temperature (X_1_, °C)	36	38	40	2
Culture time (X_2_, h)	60	72	84	12
Glucose concentration (X_3_, g/L)	5	10	15	5
pH (X_4_)	7	7.5	8	0.5

**Table 5:** Response surface experimental design scheme and results.

Number	Factors				TPC (mg GAE/mL cell-free supernatant)		TFC (mg QE/mL cell-free supernatant)	
	X_1_	X_2_	X_3_	X_4_	Observed	Predicted	Observed	Predicted
1	38	72	10	7.5	396.15±8.25	394.66	245.44±1.96	243.11
2	36	72	5	7.5	289.98±4.53	291.48	178.14±2.88	180.09
3	40	72	5	7.5	348.74±3.92	347.35	216.19±3.93	216.05
4	38	72	10	7.5	391.70±6.46	394.66	241.67±2.37	243.11
5	38	84	10	7	308.74±3.92	309.49	191.35±2.37	191.26
6	40	60	10	7.5	331.46±5.99	333.82	208.96±4.32	207.21
7	38	72	15	7	342.32±3.42	339.66	215.57±4.99	213.08
8	38	60	15	7.5	297.88±4.76	298.28	181.92±4.84	184.08
9	38	84	10	8	282.07±5.93	282.08	174.06±1.89	173.13
10	38	72	5	7	291.95±3.73	292.99	185.69±2.37	183.04
11	40	72	15	7.5	285.53±4.76	284.15	181.60±4.99	179.73
12	38	72	10	7.5	393.68±5.99	394.66	242.92±0.94	243.11
13	38	72	10	7.5	395.16±4.76	394.66	243.87±2.83	243.11
14	40	72	10	7	351.70±6.46	352.85	219.03±3.31	221.97
15	40	84	10	7.5	274.67±5.34	275.46	169.97±1.96	169.79
16	40	72	10	8	253.43±3.08	251.86	155.19±3.77	156.20
17	38	60	10	8	311.21±5.61	310.57	191.04±3.77	191.21
18	36	60	10	7.5	266.77±3.73	267.81	165.25±2.88	163.08
19	38	60	5	7.5	343.31±7.00	340.01	210.22±3.03	210.81
20	36	84	10	7.5	306.77±3.08	306.25	188.21±3.40	187.61
21	36	72	15	7.5	303.31±3.08	304.81	189.15±5.25	189.37
22	38	72	5	8	351.21±9.64	355.71	219.97±3.03	220.10
23	38	60	10	7	300.84±4.53	300.94	185.06±3.81	186.07
24	38	84	5	7.5	315.65±9.05	313.26	191.04±3.77	191.16
25	36	72	10	7	243.56±7.41	243.14	148.27±4.75	149.54
26	36	72	10	8	329.48±2.96	326.35	202.99±1.44	202.32
27	38	72	10	7.5	396.64±4.53	394.66	241.67±1.96	243.11
28	38	72	15	8	258.37±8.25	259.16	162.74±4.99	163.03
29	38	84	15	7.5	303.80±3.73	305.11	189.15±1.89	190.84

*Note:* X_1_ is culture temperature (^օ^C); X_2_ is culture time (hour); X_3_ is D-glucose concentration (g/L); X_4_ is pH.

The significance of each variable and their interactions was determined using *p*-values, which revealed that A, B, C, D, A^2^, B^2^, C^2^, and D^2^ were significant model terms. The ability of DO-R5 to synthesize polyphenols and flavonoids was significantly impacted (*p*<0.05) by interactions between temperature and time (AB), temperature and glucose concentration (AC), temperature and pH (AD), time and glucose concentration (BC), time and pH (BD), and glucose concentration and pH (CD). The correlation coefficient (R^2^) of the regression equation were 0.998 and 0.994, respectively, indicating that the obtained equation was well-fitted and could be used to predict test results. The regression model showed strong significance (*p*<0.0001), with no statistically noteworthy lack of fit terms (0.2737 for TPC and 0.2664 for TFC, p>0.05). This suggests that the model was reasonable and feasible for analyzing and predicting polyphenol and flavonoid production in the DO-R5 bacterial strain.

A three-dimensional response surface was used to demonstrate the interaction of four factors in **S.** Fig. 1. Factors A-B, A-C, A-D, B-C, B-D, and C-D all showed extremely steep surfaces, indicating that they had a significant influence on polyphenol and flavonoid synthesis of DO-R5. The optimal cultivation conditions were found to be the following: Cultivation temperature was 38 °C, cultivation time was 70.5 h, glucose concentration was 8.9 g/L glucose, and the initial pH was 7.5. Under optimum conditions, the predicted polyphenol and flavonoid values were 397.04 mg GAE/mL and 244.82 mg QE/mL, respectively. Each experiment was repeated three times to ensure experimental feasibility and appropriate culture conditions. The TPC and TFC levels were 406.52 mg GAE/mL and 248.27 mg QE/mL, respectively, which were comparable with theoretical values (p>0.05). These findings indicated that the model utilized in this investigation was reasonable and practicable, and that it could be used to anticipate the conditions for growing *Bacillus* sp. DO-R5 in order to maximize polyphenol and flavonoid production. After optimizing the culture conditions, *Bacillus* sp. DO-R5 produced considerably more TPC and TFC than normal conditions. TPC and TFC increased by 98.26 % and 70.70 %, respectively, in cell-free supernatant; 173.38 % and 167.48 %, respectively, in the extracts (Fig. 7).

### 3.6 Compound determination of DO-R5 extracts by HPLC

Analysis of polyphenol and flavonoid content and composition by HPLC showed that *Bacillus* sp. DO-R5 strain produced gallic acid (peak 1, wavelength 270 nm), chlorogenic acid (peak 2, wavelength 340 nm), vanillic acid (peak 3, wavelength 270 nm), caffeic acid (peak 4, wavelength 270 nm), rutin (peak 5, wavelength 340 nm), diosmin (peak 6, wavelength 340 nm), quercetin (peak 7, wavelength 340 nm), kaempferol (peak 8, wavelength 340 nm). Chromatograms of polyphenol compounds (gallic acid, chlorogenic acid, vanillic acid, and caffeic acid) and flavonoids (rutin, diosmin, quercetin, and kaempferol) of nDO-R5e and oDO-R5e extracts are shown in Fig. 8.

The composition and content of polyphenols and flavonoids in nDO-R5e and oDO-R5e extracts are shown in Table 6. The results showed that oDO-R5e extract had higher content of gallic acid, chlorogenic acid, vanillic acid, caffeic acid, rutin, diosmin, and quercetin than that of nDO-R5e extract by 6.17, 4.89, 2.13, 3.11, 6.39, 15.10, and 1.23 times, respectively. In addition, kaempferol compound also appeared in oDO-R5e extract with a content of 1.65±0.01 µg/mg extract (Table 7).

**Table 6:** Variance analysis of TPC and TFC production using the regression model.

Source	df	Sum of Squares		Mean Square		F-value		*p*-value	
		TPC	TFC	TPC	TFC	TPC	TFC	TPC	TFC
**Model**	14	56929.35	21598.58	4066.38	1542.76	604.16	356.06	<0.0001	<0.0001
**A**	1	930.34	519.16	930.34	519.16	138.22	119.82	<0.0001	<0.0001
**B**	1	297.70	124.61	297.70	124.61	44.23	28.76	<0.0001	0.0001
**C**	1	1865.76	548.37	1865.76	548.37	277.20	126.56	<0.0001	<0.0001
**D**	1	237.10	126.62	237.10	126.62	35.23	29.22	<0.0001	<0.0001
**AB**	1	2342.08	959.45	2342.08	959.45	347.97	221.44	<0.0001	<0.0001
**AC**	1	1464.59	519.84	1464.59	519.84	217.60	119.98	<0.0001	<0.0001
**AD**	1	8481.49	3514.12	8481.49	3514.12	1260.13	811.05	<0.0001	<0.0001
**BC**	1	281.90	174.37	281.90	174.37	41.88	40.24	<0.0001	<0.0001
**BD**	1	342.99	135.37	342.99	135.37	50.96	31.24	<0.0001	<0.0001
**CD**	1	5127.28	1897.04	5127.28	1897.04	761.78	437.83	<0.0001	<0.0001
**A^2^**	1	18236.07	6664.29	18236.07	6664.29	2709.42	1538.10	<0.0001	<0.0001
**B^2^**	1	13607.11	5509.16	13607.11	5509.16	2021.67	1271.50	<0.0001	<0.0001
**C^2^**	1	7806.41	2530.01	7806.41	2530.01	1159.84	583.92	<0.0001	<0.0001
**D^2^**	1	14999.45	5287.89	14999.45	5287.89	2228.54	1220.43	<0.0001	<0.0001
**Residual**	15	94.23	60.66	6.73	4.33				
**Lack of Fit**	10	78.12	50.47	7.81	5.05	1.94	1.98	0.2737	0.2664
**Pure Error**	5	16.11	10.19	4.03	2.55				
**Cor Total**	29	57023.58	21659.24						
**R^2^**		0.998	0.997						
**R^2^_Adjusted_**		0.997	0.994						
**R^2^_Predicted_**		0.992	0.986						
**CV%**		0.81	1.05						
**N**		29	20

**Table 7:** Polyphenol and flavonoid compounds present in nDO-R5e and oDO-R5e extracts.

**Compound**	**Content (µg/mg extract)**	
	nDO-R5e	oDO-R5e
Gallic acid	3.07^b^±0.12	18.93^a^±0.93
Chlorogenic acid	6.84^b^±0.08	33.47^a^±1.33
Vanillic acid	7.62^b^±0.03	16.20^a^±1.27
Caffeic acid	7.54^b^±0.05	23.43^a^±0.98
Rutin	7.01^b^±0.79	44.77^a^±0.21
Diosmin	16.18^b^±2.52	244.31^a^±8.24
Quercetin	7.33^b^±0.39	8.98^a^±0.27
Kaempferol	-	1.65±0.01

*Note:* Different letters in the same row show significant difference at the level of 5 % (*p*<0.05). “-” Means not detected.

### 3.7 *In vitro* antioxidant, anti-inflammatory, α-amylase and α-glucosidase inhibitory activities of *Bacillus* sp. DO-R5 extracts

**Fig. 6:**
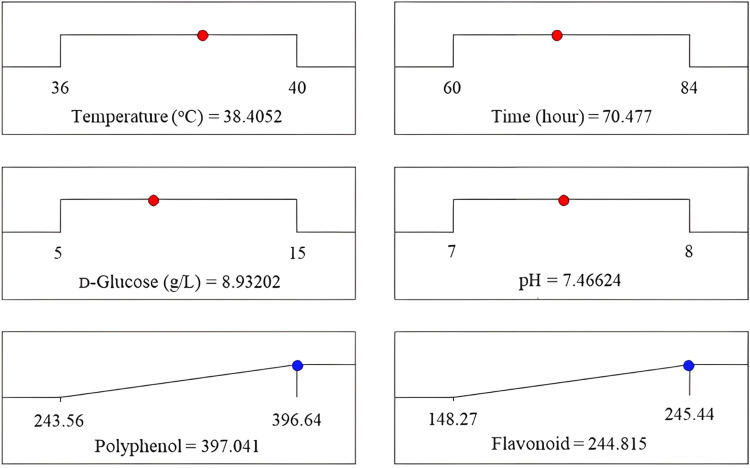
The model predicts optimal culture conditions for the DO-R5 bacterial strain with the ability to produce polyphenols and flavonoids.

*Bacillus* sp. DO-R5 was cultured under two conditions. For the normal condition, the strain was grown in 1 L PDB medium (20 g/L glucose, 30 °C, pH 7.0, shaking at 200 rpm) for 24 h. For the optimal condition, the strain was grown in 1 L PDB medium (8.9 g/L glucose, 38 °C, pH 7.5, shaking at 200 rpm) for 70.5 h (Fig. 6). Each condition was scaled up by performing multiple 1 L shake-flask cultures to reach a total culture volume of 100 L. The pooled cultures were extracted, yielding 20.67 g of crude extract (nDO-R5e) under normal conditions and 32.45 g of crude extract (oDO-R5e) under optimal conditions. TPC and TFC in oDO-R5e extract (TPC=590.22 mg GAE/g extract; TFC=436.95 mg QE/g extract) were 2.73 and 2.67 times higher than those in nDO-R5e extract (TPC=215.90 mg GAE/g extract; TFC=163.36 mg QE/g extract), respectively. The antioxidant activity of DO-R5 extract was determined by ABTS^•+^, DPPH, FRAP, TAC and NO^•^ methods, and the antioxidant effect was expressed as the 50 % inhibition concentration (IC_50_) of free radicals. oDO-R5e extract showed very high antioxidant activity with IC_50_<50 µg/mL against ABTS^•+^ (IC_50_=17.41 µg/mL), DPPH (IC_50_=15.27 µg/mL), FRAP (IC_50_=21.15 µg/mL), TAC (IC_50_=28.55 µg/mL) and NO^•^ (IC_50_=45.41 µg/mL) (Table 8). oDO-R5e extract effectively inhibited α-amylase and α-glucosidase, with IC_50_ values ​​of 29.48 and 10.78 µg/mL, respectively. Very small IC_50_ means strong inhibitory activity. oDO-R5e extract significantly inhibited the activities of α-amylase and α-glucosidase enzymes, as indicated by low IC_50_ values ​​(Table 9).

**Fig. 7:**
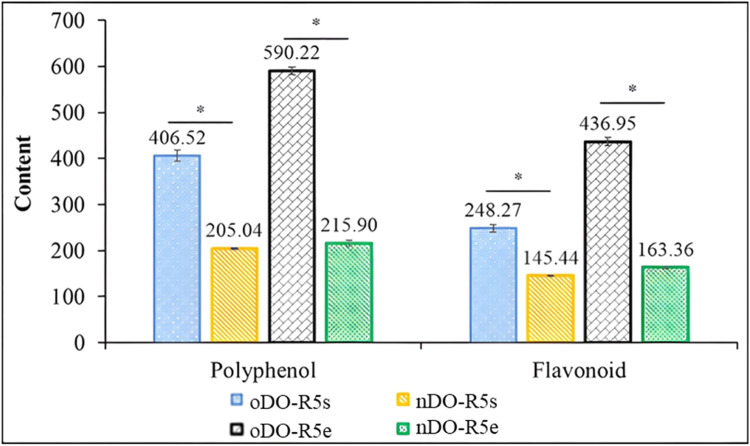
Polyphenol and flavonoid contents in cell-free supernatant and extracts of Bacillus sp. DO-R5 under normal and optimal culture conditions.

Notes: nDO-R5s – cell-free supernatant under normal conditions; oDO-R5s – cell-free supernatant under optimal conditions; nDO-R5e – extract from cell-free supernatant under normal conditions; oDO-R5e – extract from cell-free supernatant under optimal conditions. Polyphenols are expressed as mg gallic acid equivalents (GAE) per mL (cell-free supernatant) or mg GAE per g (extract); flavonoids as mg quercetin equivalents (QE) per mL (cell-free supernatant) or mg QE per g (extract). *p<0.05.

**Table 8:** *In vitro* biological activity of DO-R5 extracts.

**Biological activity**	**Methods**	**The IC_50_ value (µg/mL)**		
		nDO-R5e	oDO-R5e	**Standards**
Antioxidant	ABTS^•+^	50.45^a^±0.87	17.41^b^±0.11	5.85^c^±0.31
	DPPH	44.66^a^±0.32	15.27^b^±0.14	4.13^c^±0.13
	FRAP	69.69^a^±0.19	21.15^b^±0.45	10.37^c^±0.31
	TAC	92.08^a^±1.59	28.55^b^±1.46	17.86^c^±1.01
	NO^•^	134.16^a^±0.79	45.41^b^±1.49	21.86^c^±0.53
Anti-inflammatory	BSA	70.00^a^±1.89	26.26^b^±1.15	22.17^c^±0.45
	RBCs	66.80^a^±0.63	23.61^b^±1.36	17.67^c^±0.68
Anti-diabetic	α-Amylase	113.78^a^±2.79	29.48^b^±1.08	14.07^c^±0.09
	α-Glucosidase	43.79^a^±0.74	10.78^b^±0.15	7.72^c^±0.10

*Note:* Different letters in the same row show sinificant difference at the level of 5 % (*p*<0.05). Ascorbic acid, diclofenac and acarbose was used as standard for antioxidant, anti-inflammatory, anti-diabetic acitivities, respectively. nDO-R5e and oDO-R5e are the *Bacillus* sp. DO-R5 extracts under normal and optimal culture conditions.

**Table 9:** Body weight and the biochemical parameters of mice under different treatments.

**Parameters**	**Groups**					
	**N**	**D**	**D, Gli**	**D, oDO-R5e, 100**	**D, oDO-R5e, 200**	**D, oDO-R5e, 400**
Body weight (g)	36.71^a^±1.34	16.94^d^±1.44	36.70^a^±1.30	31.76^c^±1.02	33.90^b^±0.80	35.73^ab^±0.93
Glucose (mg/dL)	116.23^c^±4.97	403.46^a^±23.13	194.40^b^±16.56	203.40^b^±36.58	189.77^b^±15.23	142.97^c^±7.34
Cholesterol (mg/dL)	130.72^c^±5.19	288.58^a^±20.36	120.79^c^±27.37	166.57^b^±12.09	138.44^c^±9.83	137.89^c^±8.26
Triglyceride (mg/dL)	98.61^bc^±20.07	722.76^a^±37.34	121.37^b^±17.49	101.14^bc^±20.35	78.38^c^±14.83	75.85^c^±19.69
LDL-C (mg/dL)	76.80^b^±5.24	138.63^a^±18.03	67.29^b^±23.90	116.56^a^±10.20	84.16^b^±14.22	75.84^b^±9.56
VLDL-C (mg/dL)	19.72^bc^±4.01	144.55^a^±7.47	24.27^b^±3.50	20.23^bc^±4.07	15.68^c^±2.97	15.17^c^±3.94
HDL-C (mg/dL)	34.20^c^±5.19	5.41^d^±0.83	29.23^c^±5.40	29.79^bc^±8.84	38.61^ab^±5.90	46.88^a^±4.13
AIP	0.46^bc^±0.15	2.13^a^±0.06	0.62^b^±0.10	0.54^b^±0.14	0.31^cd^±0.13	0.20^d^±0.13
AC	2.91^b^±0.68	53.09^a^±5.56	3.14^b^±0.58	4.98^b^±1.56	2.70^b^±0.91	1.97^b^±0.36
CRR	3.91^b^±0.68	54.09^a^±5.56	4.14^b^±0.58	5.98^b^±1.56	3.70^b^±0.91	2.97^b^±0.36
CPI	0.45^b^±0.08	0.04^d^±0.00	0.46^ab^±0.10	0.26^c^±0.10	0.48^ab^±0.16	0.63^a^±0.13
ALT (U/L)	35.00^d^±3.56	496.86^a^±23.68	50.14^d^±5.08	358.57^b^±12.15	159.86^c^±8.38	46.86^d^±5.55
AST (U/L)	58.00^d^±2.52	604.00^a^±46.60	76.43^d^±4.76	441.43^b^±28.54	176.57^c^±7.61	65.00^d^±4.24
Urea (mg/dL)	33.73^e^±1.59	116.95^a^±2.52	40.02^d^±2.05	95.09^b^±2.24	53.61^c^±2.76	35.89^e^±3.05
Creatinine (µmol/L)	69.29^e^±3.50	258.43^a^±5.26	87.00^d^±3.83	153.29^b^±7.95	107.71^c^±5.28	75.57^e^±3.74

*Note:* Values are presented as means ± SE. The number of mice in each group was 7. N: Normal mice treated with water; D: Non-treated diabetic mice; D, Gli: Glibenclamide treated-diabetic mice at the dose 10 mg/kg; D, oDO-R5e 100, D, oDO-R5 200e, D, oDO-R5e 400: The oDO-R5e extract-treated diabetic mice at the dose of 100, 200, and 400 mg/kg, respectively. Different letters in the same row show sinificant difference at the level of 5 % (*p*<0.05).

The anti-inflammatory activity of oDO-R5e extract is shown in Table 8. Diclofenac was used as a control. oDO-R5e inhibited protein denaturation and protected red blood cells, with IC_50_ values ​​of 26.26 and 23.61 µg/mL, respectively.

Table 8 also compared the antioxidant, anti-inflammatory, α-amylase, and α-glucosidase inhibitory activities of *Bacillus* sp. DO-R5 extract under normal (abbreviated as nDO-R5e) and optimal (abbreviated as oDO-R5e) conditions. The statistics showed that all the bioactivities evaluated above were significantly increased.

### 3.8 Effects of DO-R5 extract on alloxan monohydrate induced diabetic mice

Body weight and biochemical parameters of mice under different treatments are presented in Table 9. In untreated diabetic mice (D), body weight decreased by more than 50 %, while blood glucose levels increased dramatically (approximately 2.8 times) when compared to normal mice (N). Diabetic mice treated with glibenclamide (D, Gli) and DO-R5 extract at various doses showed significant weight gain as compared to non-treated diabetic mice (*p*<0.05). Diabetic mice's blood glucose levels reduced dramatically in in a dose-dependent with the 400 mg/kg group being the most effective (D, DO-R5, 400) that had a similar blood glucose level to the normal group. Noticeably, the effectiveness of the 200 mg/kg dose (D, DO-R5, 200) in lowering blood glucose levels was equivalent to that of the commercial drug (D, Gli), although the 400 mg/kg dose (D, DO-R5, 400) reduced blood glucose levels more significantly.

Biochemical indexes of TC, TG, LDL-C, VLDL-C, AIP, AC, CRR, AST, ALT, urea, and creatinine significantly increased in the D group when compared to those of normal mice (N) (*p*<0.05). These parameters were decreased after the treatment of glibenclamide or oDO-R5e. On the other hand, CPI and HDL-C were lower in D group than in N group. The treatment of glibenclamide or oDO-R5e extract increased CPI value in diabetic mice. TC, TG, LDL-C, VLDL-C, AIP, AC, CRR, CPI, and HDL-C responded to oDO-R5e treatment in a dose-dependent manner. Remarkably, diabetic mice treated with oDO-R5e 400 mg /kg had body weight and nearly all biochemical parameters analyzed that were equivalent to those of normal mice (N).

Untreated diabetic mice significantly increased MDA (lipid peroxidation product) and reduced the GSH (endogenous antioxidant) level in tissues of all test organs (liver, kidneys, and pancreas) as compared to those of normal mice (Table 9). Diabetic mice treated with oDO-R5e showed dose-dependent reductions in MDA and increases in GSH in all examined organs. MDA levels in mice treated with 400 mg/kg were lower, while GSH levels were higher than in the normal and glibenclamide-treated diabetic groups in all investigated organs.

Histochemical staining images of the liver, kidneys, and pancreas from different treatment groups are presented in Figs. 9, 10, and 11. Cellular and nuclear degradations are mainly evidence of organ damage. The untreated diabetic mice (D group) exhibited abnormal structural changes in tested organs. The liver contained an accumulation of lipid droplets (Fig. 9**B**). The kidneys displayed a variety of changes, including glomerular degeneration, tubulointerstitial wounds, glomerular sclerosis, vacuolation of tubular epithelial cells, and loss of the brush boundary (Fig. 10**B**), as well as islet atrophy and pancreatic central venous congestion.

(Fig. 11**B**). Notably, administration of oDO-R5e at doses of 100, 200, and 400 mg/kg effectively prevented the severe hepatic, renal, and pancreatic damage caused by alloxan monohydrate. The tissue structures in the 400 mg/kg treatment group closely resembled those of the normal (N) group, suggesting that oDO-R5e confers protective effects against the acute toxicity induced by AM.

## 4 Discussion

Polyphenols and flavonoids have been found to work as antioxidants, preventing major diseases such as diabetes, liver damage, arthritis, cardiovascular disease, and aging [40]. In this work, 19 endophytic bacterial strains isolated from DO roots, stems, and leaves were evaluated for TPC and TFC. DO-R5 strain produced the greatest TPC and TFC levels, 206.52 mg GAE/mL and 144.81 mg QE/mL, respectively. Endophytic bacteria can create TPC and TFC, which have antioxidative and anti-inflammatory properties [9,10,12,41]. They also block the activities of α-amylase and α-glucosidase [[23], [24], [25]]. The polyphenol group, which includes compounds like gallic acid, chlorogenic acid, vanillic acid, and caffeic acid, is notable for its potent antioxidant qualities because of its structure, which includes several hydroxyl groups that aid to neutralize free radicals and lessen oxidative stress. Gallic acid and caffeic acid shield cell membranes from oxidative damage by lowering lipid peroxidation and suppressing the production of free radicals [42]. By blocking the α-glucosidase enzyme and delaying the absorption of glucose, chlorogenic acid and vanillic acid also play a major role in lowering blood glucose levels and aiding in the management of diabetes [43]. Besides, polyphenols can control inflammation by suppressing cytokines like TNF-α and IL-6 [44]. The flavonoid family, which includes rutin, diosmin, quercetin, and kaempferol, is significant for both antioxidant and anti-inflammatory properties. Quercetin and kaempferol have been shown to suppress inflammatory enzymes such as COX-2 and lipoxygenase while decreasing the formation of nitric oxide, a component that causes inflammation [45]. Rutin and diosmin are very efficient in protecting vessel walls and reducing phlebitis due to their method of increasing vascular strength and decreasing capillary permeability [46]. Flavonoids in diabetes enhance insulin sensitivity, reduce resistance, and protect pancreatic β-cells from oxidative stress [47,48]. The combination of polyphenols and flavonoids promotes the production of antioxidant, anti-inflammatory, and anti-diabetic properties.

Although there were many different types of endophytic bacteria, *Bacillus, Pantoea*, and *Pseudomonas* were the most common genera. In this study, *Bacillus* sp. DO-R5 strain was found as the strain with the highest TPC and TFC production. This conclusion was consistent with prior research, which demonstrated that *Bacillus* can create antioxidants [49,50]. DO, a plant species that commonly grows wild in Vietnam, inhibits α-amylase and α-glucosidase, has antioxidant capacities, and contains phenolic compounds with drug-like effects [7]. *Bacillus* sp. DO-R5 isolated from DO may provide the same benefits as the host plants. Previous studies shown a linear correlation of total polyphenol and flavonoid concentration to the biological activities, specifically antioxidant [[51], [52], [53]], anti-inflammatory [51,54,55], and anti-diabetic [49,51]. Polyphenols and flavonoids were used as indicators to optimize the *Bacillus* sp. DO-R5 cultivation conditions. The ideal incubation temperature, duration, glucose concentration, and pH were discovered to be 38 °C, 70.5 h, 8.9 g/L D-glucose, and pH 7.5. Many *Bacillus* species produced high-benefit metabolites under conditions comparable to those used in this study [56,57], indicating *Bacillus* sp. DO-R5 developed and maximized secondary metabolites in the experimental conditions.

Oxidative stress has frequently been linked to the development of hyperglycemia and related problems [58]. Furthermore, inflammation is thought to be an essential indicator of type 2 diabetes and insulin resistance, as there is a link between elevated levels of circulating acute-phase inflammatory markers, insulin resistance indices, and diabetes progression [59]. As a results, in recent years, academic and pharmaceutical researchers have paid close attention to the antidiabetic capabilities of natural sources, which include antioxidant, anti-inflammatory, and hypoglycemic effects. Endophytic bacteria can produce metabolitic compounds, which have emerged as a new source in biomedicine.

The cell-free supernatant of *Bacillus* sp. DO-R5 growth in optimal culture conditions was extracted with ethyl acetate. *In vitro*, oDO-R5e was tested for anti-oxidative, anti-inflammatory, and enzyme inhibition properties, including α-amylase and α-glucosidase. During this study, we observed that the evaluation of anti-inflammatory activity as well as inhibition of α-amylase and α-glucosidase enzymes is commonly based on IC_50_ values and comparison with standard reference compounds such as acarbose (for enzyme inhibition), diclofenac (for anti-inflammatory activity). Although no absolute international standard has been established, based on literature synthesis and research experience, we propose the following classification of anti-inflammatory and α-amylase/α-glucosidase inhibitory activities according to IC_50_ values: very strong activity when IC_50_ < 10 µg/mL (or < 10 µM for pure compounds), strong activity when IC_50_ is between 10–50 µg/mL (or 10–50 µM), moderate activity when IC_50_ is between 50–100 µg/mL (or 50–100 µM), and weak activity when IC_50_ > 100 µg/mL (or > 100 µM). For crude extracts, the thresholds may be more flexible; however, in general, the lower the IC_50_, the stronger the activity. This classification is similar to that used in other bioactivity assessments, such as antioxidant activity, in which IC_50_ < 10 µg/mL is considered very strong, 10–50 µg/mL is strong, 50–100 µg/mL is moderate, and >100 µg/mL is weak [60,61]. Applying these thresholds allows for an objective comparison of the effectiveness of test samples with standard reference compounds and supports the selection of potential candidates for further investigation. This work used a variety of assays to discover the antioxidant properties of the oDO-R5e from cell-free supernatant of *Bacillus* sp. DO-R5 strain. The human body contains numerous oxidants that were formed by certain processes. The values of IC_50_ for both nDO-R5e and oDO-R5e were less than 50 μg/mL (Table 8), indicating that oDO-R5e possessed high antioxidant properties [62].

When compared to diclofenac, oDO-R5e dramatically reduced protein bovine serum albumin denaturation and stabilized erythrocyte membranes against hypotomicity-induced hemolysis (Table 8). These data prove that oDO-R5e may include anti-inflammatory capabilities. It has been showed that anti-inflammatory extracts can reduce protein denaturation, while protecting cell membranes from lysis [30,31]. Protein denaturation is a biological marker of chronic inflammatory processes and can lead to dysfunction of tissue.

[63]. Lysosomal membrane disruption during chronic inflammation has also been demonstrated, resulting in mediators of inflammation such as neutrophil filtration, proteases, and histamine release near the site of tissue injury [64]. Therefore, oDO-R5e, which shields the cell membrane from lysis and stops protein denaturation, could be a promising source of anti-inflammatory medications.

Alpha-amylase, an enzyme, is responsible for hydrolyzing starch to disaccharide and oligosaccharide, while α-glucosidase breaks down disaccharide into glucose. The inhibition or delay of these enzymes controls the blood glucose level due to preventing the breakdown of carbohydrates to glucose. oDO-R5e had a significant (IC_50_ < 50 mg/mL) effect on α-amylase and α-glucosidase inhibitory activity (Table 8). Polyphenols and flavonoids not only reduce oxidative stress but also inhibit carbohydrate-hydrolyzing enzymes to prevent hyperglycemia [65,66]. *Bacillus* sp. DO-R5 generate large levels of polyphenols and flavonoids, a source for antioxidative and inflammatory capacities. It also inhibits α-amylase and α-glucosidase activity, making it a promising therapeutic candidate.

Alloxan has been frequently used to cause diabetes in animals due to its ability to activate free radical generation via redox processes [67], which results in oxidative stress and diabetes. In this study, when non-treated diabetic mice (D group) were subjected to AM, their blood glucose levels were considerably higher and their body weight was lower than in normal mice (N group). Diabetes has two major characteristics: Hyperglycemia and weight loss. When compared to untreated diabetic mice, oral treatment of oDO-R5e showed a significant decrease in blood glucose levels and increase in weight growth (*p*<0.05) (Table 9). Remarkably, the body weight and blood glucose level in mice under 400 mg/kg BW oDO-R5e treatment were comparable to those of normal mice. Glibenclamide treatment could decrease blood glucose in diabetic mice to a level that was still significantly higher than that of normal mice. In diabetes, chronic hyperglycemia contributes to increased ROS production, aggravating oxidative stress [68]. Table 10 showed that MDA, the product of lipid peroxidation, level in the liver, kidneys, and pancreas of alloxan-induced diabetic mice was about 4.5, 5.4 and 6 times higher than those of normal mice, respectively. While GSH, an endophytic antioxidant, level in the organs of alloxan-induced diabetic mice was lower than those of normal mice 8, 5, and 4 times in liver, kidneys and pancreas, respectively. The results showed that AM disrupted the balance between oxidant and antioxidant in diabetic mice, as well as high chronic glucose levels, resulting in excessive ROS production.

**Table 10:** MDA and GSH content of test mices under different treatment.

**Groups**	**MDA (nM MDA/g tissue)**			**GSH (nM GSH/g tissue)**		
	**Liver**	**Kidneys**	**Pancreas**	**Liver**	**Kidneys**	**Pancreas**
N	16.37^e^±1.20	12.86^e^±0.97	10.95^e^±0.48	239.98^b^±7.99	251.36^c^±1.85	261.58^c^±2.25
D	73.86^a^±0.93	70.26^a^±0.62	60.74^a^±0.64	29.76^e^±1.85	50.70^f^±2.09	60.59^f^±2.49
D, Gli	21.11^d^±0.57	16.44^d^±0.61	12.64^d^±0.80	258.78^a^±4.36	269.17^b^±2.96	302.14^b^±2.96
D, oDO-R5e, 100	55.40^b^±1.51	53.68^b^±1.06	42.80^b^±0.92	52.02^d^±1.47	81.53^e^±1.55	105.61^e^±1.49
D, oDO-R5e, 200	32.45^c^±0.94	31.98^c^±0.77	30.53^c^±0.69	210.64^c^±2.98	231.08^d^±2.08	241.47^d^±3.63
D, oDO-R5e, 400	14.30^f^±1.11	11.35^f^±0.49	8.91^f^±1.20	259.61^a^±1.13	284.17^a^±2.96	293.74^a^±2.00

*Note:* Values are presented as means ± SE. The number of mice in each group was 7. N: Normal mice treated with water; D: Non-treated diabetic mice; D, Gli: Glibenclamide treated-diabetic mice at the dose 10 mg/kg; D, oDO-R5e 100, D, oDO-R5e 200, D, oDO-R5e 400: The oDO-R5e extract-treated diabetic mice at the dose of 100, 200, and 400 mg/kg, respectively. Different letters in the same column show sinificant difference at the level of 5 % (*p*<0.05).

Diabetes mellitus has been associated to lipid abnormalities such as hypercholesterolemia and hypertriglyceridemia, which are characterized by significant increases in TG, TC, LDL-C, and VLDL-C levels, as well as low HDL-C concentrations in serum. In diabetic mice treated with oDO-R5e, triglyceride, cholesterol, LDL-C, and VLDL-C levels were significantly lower, but HDL-C levels were higher (Table 9). LDL-C is regarded a substantial risk factor for revascularization, ischemic strokes, atherothrombotic processes, and cardiovascular mortality [69]. Elevated levels of TG and cholesterol were found to increase the risk of cardiovascular diseases (CVD) which related to increased LDL-C [70]. HDL-C is considered to be “good” for the circulatory system, as it was consistently demonstrated to reduce the risk of CVD on clinical and epidemitological studies. The lower value of CPI is associated with a higher risk of CVD. CPI was very low in the non-treated diabetic group, but significantly increased in oDO-R5e treated groups to values that were higher than in normal mice (Table 9). In contrast, a higher value of the AIP, AC, or CRR implies a greater risk of CVD. The AIP, AC, and CRR values in the non-treated diabetic group were 4.6, 18.2, and 13.8 times higher than in the normal group, respectively, whereas oDO-R5e treated animals exhibited lower values than normal mice (Table 9). The outcomes showed that oDO-R5e had a strong cardiovascular protective effect on diabetic mice.

**Fig. 8:**
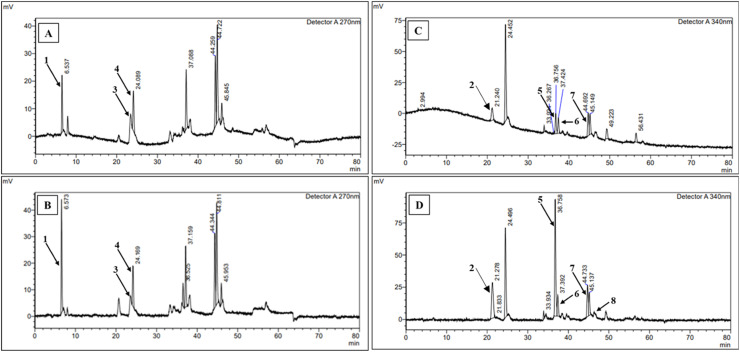
Liquid chromatographic profiles of nDO-R5e and oDO-R5e extracts.

Note: A, C are the liquid chromatographic profiles of nDO-R5e extracts detected at 270 nm and 340 nm wavelengths, respectively; B, D are the liquid chromatographic profiles of oDO-R5e extracts detected at 270 nm and 340 nm wavelengths, respectively. 1: gallic acid, 2: chlorogenic acid, 3: vanillic acid, 4: caffeic acid, 5: rutin, 6: diosmin, 7: quercetin, 8: kaempferol.

**Fig. 9:**
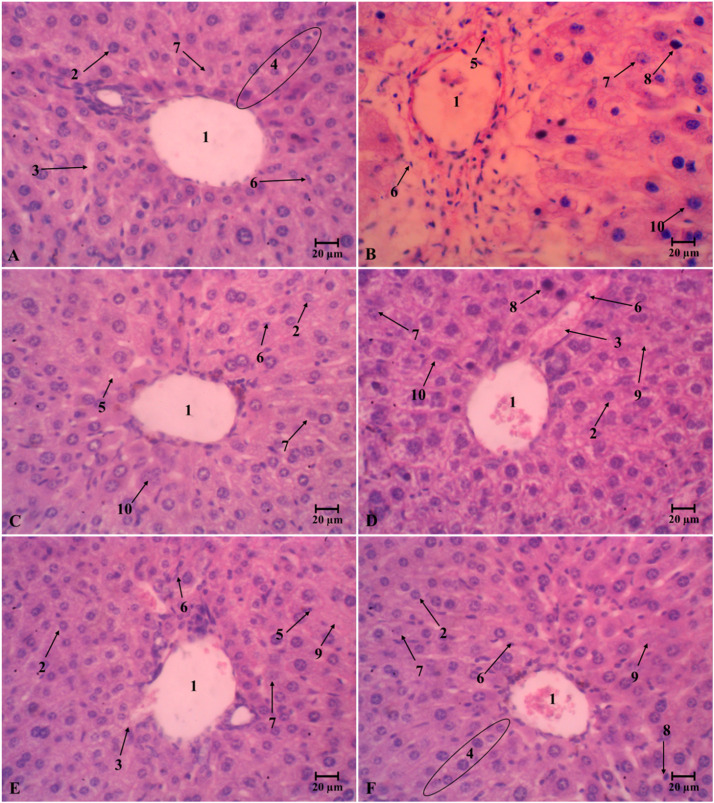
Light microscopic photographs of representative liver section at 400X magnification.

Note: A-Normal mice drink water; B-non-treated diabetic mice; C-Glibenclamide-treated diabetic mice (dose 10 mg/kg); D-The oDO-R5e extract-treated diabetic mice (dose 100 mg/kg); E-The oDO-R5e extract-treated diabetic mice (dose 200 mg/kg); F-The oDO-R5e extract-treated diabetic mice (dose 400 mg/kg). 1: Central vein, 2: Normal hepatocytes, 3: Sinusoids, 4: Sheets of hepatocyte, 5: Monocytes, 6: Kupffer cell, 7: Bile duct, 8: Pyknotic nucleus, 9: Binuclear hepatocyte, 10: Hepatocellular necrosis.

**Fig. 10:**
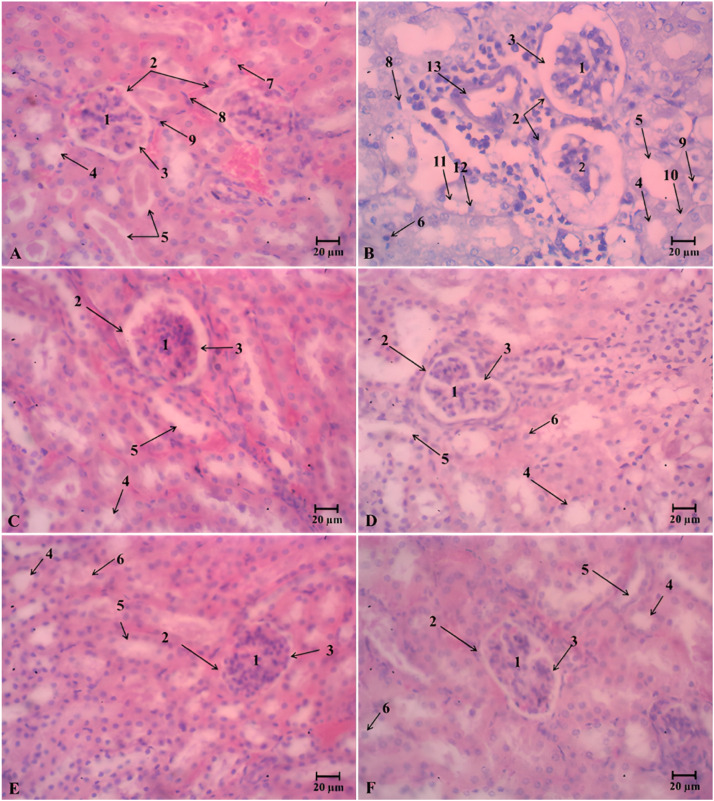
Light microscopic photographs of representative kidneys section at 400X magnification.

Note: A-Normal mice drink water; B-non-treated diabetic mice; C-Glibenclamide-treated diabetic mice (dose 10 mg/kg); D-The oDO-R5e extract-treated diabetic mice (dose 100 mg/kg); E-The oDO-R5e extract-treated diabetic mice (dose 200 mg/kg); F-The oDO-R5e extract-treated diabetic mice (dose 400 mg/kg). 1: Malpighi capillaries, 2: Glomerulus, 3: Bowman, 4: Proximal convoluted tubule, 5: Distal convoluted tubule, 6: Binuclear cell, 7: Normal cell, 8,9: Macrophage cell, 10: Renal interstitium, 11: Necrosis, 12: Pyknotic nucleus, 13: Arteries.

**Fig. 11:**
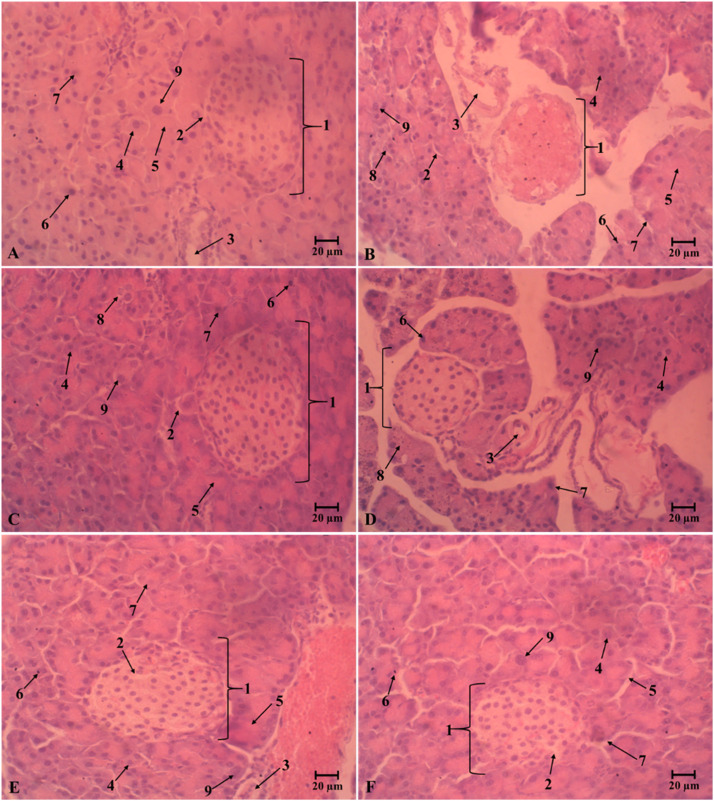
Light microscopic photographs of representative pancreas section at 400X magnification.

Note: A-Normal mice drink water; B-non-treated diabetic mice; C-Glibenclamide-treated diabetic mice (dose 10 mg/kg); D-The oDO-R5e extract-treated diabetic mice (dose 100 mg/kg); E-The oDO-R5e extract-treated diabetic mice (dose 200 mg/kg); F-The oDO-R5e extract-treated diabetic mice (dose 400 mg/kg). 1: Islet of Langerhans, 2: Blood vessels, 3: Interlobular ducts, 4: Normal cell, 5: Necrosis, 6: Binuclear cell, 7: Pyknotic nucleus, 8: Macrophage cell, 9: Hypertrophy, 10: Pancreatic cyst. 11: Lipid droplets.

The enhanced generation of ROS and oxidative stress in diabetic animals could cause damage of liver, kidneys, and pancreas [[71], [72], [73]]. Alloxan-induced diabetes in non-treated mice (D group) was closely correlated to hepatic and kidneys damage that were characterized by elevating in AST, ALT, urea and creatinine (Table 9). As the liver and kidneys are vital organ for glucose and lipid metabolism. However, serum levels of these markers in diabetic mice significantly decreased after oDO-R5e or glibenclamide treatment. Particularly, diabetic mice under the treatment of oDO-R5e 400 mg/kg BW obtained the same levels of these markers as reported in normal mice. The results indicated oDO-R5e possesses great liver and kidneys protection activities. Figs. 8, 9, and 10 reveal that alloxan-induced diabetic mice have severe deterioration in nearly all liver, kidney, and pancreatic tissues (Figs. 9**B,** 10**B, and** 11**B**). The results indicated the effect of the diabetic state in the tissues of mice induced by alloxan monohydrate. The pathological alterations were associated with hyperglycemia and inflammation processes in diabetic mice. Nevertheless, diabetic mice treated with oDO-R5e at dose of 400 mg/kg BW significantly restored hepatocellular texture with the central vein, distal, proximal tubular, and glomerulus structure (Fig. 9**F**) to normal state as well as prevent atrophy of islets (Fig. 11**F**), cytoplasmic vacuolation, and pyknotic (Fig. 10**F**) role against the adverse effect of oxidative stress induced by alloxan monohydrate on liver, kidneys, and pancreas.

## 5 Conclusion

Endophytic bacteria from *Dillenia ovata* were effectively isolated, with the strain *Bacillus* sp. DO-R5 providing the most potent antioxidant, anti-inflammatory, and anti-diabetic substances *in vitro*. The culture conditions were optimized using RSM (BBD), with the ideal parameters being 8.9 g/L glucose, pH 7.5, temperature 38 °C, and culture duration of 70.5 h. *Bacillus* sp. DO-R5 strain cultured under optimal conditions increased the production of gallic acid, chlorogenic acid, vanillic acid, caffeic acid, rutin, diosmin, quercetin, and kaempferol. Extracts from *Bacillus* sp. DO-R5 cultured under optimal conditions were able to support diabetes control and its complications in experimental mice. Histopathological analysis of damaged tissues in the liver, kidney, and pancreas also revealed positive recovery. *Bacillus* sp. DO-R5 initially demonstrated the ability to produce secondary substances useful in the treatment of diabetes and its consequences.

## Funding

The authors declare that no funds, grants, or other support were received during the preparation of this manuscript.

## Ethics approval

This study was performed in line with the principles of the Declaration of Helsinki. Approval was granted by the Ethics Committee of Can Tho University (Date: 2024/11/25/No: CTU-AEC24017).

## CRediT authorship contribution statement

**Linh Chi Tran:** Writing – review & editing, Validation, Supervision, Methodology, Formal analysis. **Chong Kim Thien Duc:** Validation, Supervision, Methodology, Formal analysis. **Tuan Trong Nguyen:** Validation, Investigation. **Duy Toan Pham:** Resources, Methodology, Formal analysis. **Danh Thai Luu:** Resources, Methodology, Formal analysis. **Trang Thi Xuan Dai:** Writing – original draft, Software, Resources, Methodology, Formal analysis, Conceptualization.

## Declaration of competing interest

The authors declare no conflicts of interest. The funders had no role in the design of the study; in the collection, analyses, or interpretation of data; in the writing of the manuscript; or in the decision to publish the results.

## Data Availability

The datasets generated during and/or analysed during the current study are available from the corresponding author on reasonable request.

## Appendix Supplementary materials

Image, application 1
