# Supplementary material for: Investigating a new Dillenia ovata endophytic bacteria to produce antioxidants and anti-diabetes activity in vitro and in vivo
Source: Biotechnol Rep (Amst). 2025 Aug 20;48:e00921. doi: 10.1016/j.btre.2025.e00921 (PMC12684764; doi:10.1016/j.btre.2025.e00921)
Supplement: Supplementary file 1 [file mmc1.docx]

S. Table 1. Colony and cell morphological characteristics of endophytic bacterial strains

| **No.** | **Bacterial strain** | **Colony shape** | **Colony color** | **Elevation** | **Margin** | **Colony size (mm)** | **Cell shape** | **Gram** |
| --- | --- | --- | --- | --- | --- | --- | --- | --- |
| 1 | DO-L1 | Circular | Dark yellow | Flat | Entire | 1-1.5 | Rod- | Negative |
| 2 | DO-L2 | Circular | Milk-white | Convex | Entire | 1.5-2 | Rod-shaped | Negative |
| 3 | DO-L3 | Circular | Milk-white | Convex | Entire | 1.5-2 | Rod-shaped | Negative |
| 4 | DO-L4 | Circular | Ivory | Convex | Entire | 0.5-1 | Rod-shaped | Negative |
| 5 | DO-L5 | Circular | Milk-white | Convex | Entire | 1.5-2 | Rod-shaped | Negative |
| 6 | DO-L6 | Circular | Milk-white | Convex | Entire | 1.5-2 | Rod-shaped | Negative |
| 7 | DO-R1 | Circular | Ivory | Convex | Entire | 0.5-1 | Rod-shaped | Negative |
| 8 | DO-R2 | Circular | Ivory | Convex | Entire | 1.5-2 | Rod-shaped | Negative |
| 9 | DO-R3 | Circular | Ivory | Convex | Entire | 1.5-2 | Rod-shaped | Negative |
| 10 | DO-R4 | Circular | Off white | Flat | Entire | 1.5-2 | Rod-shaped | Negative |
| 11 | DO-R5 | Irregular | Off white | Flat | Undulate | 0.5-1.5 | Rod-shaped | Positive |
| 12 | DO-R6 | Circular | Off white | Flat | Entire | 0.5-1 | Rod-shaped | Negative |
| 13 | DO-R7 | Circular | Off white | Flat | Entire | 0.5-1 | Rod-shaped | Negative |
| 14 | DO-R8 | Circular | Off white | Flat | Entire | 1-1.5 | Rod-shaped | Negative |
| 15 | DO-R9 | Circular | Dark yellow | Flat | Entire | 0.5-1 | Rod-shaped | Positive |
| 16 | DO-S1 | Circular | Light yellow | Flat | Entire | 1-1.5 | Rod-shaped | Negative |
| 17 | DO-S2 | Circular | Light yellow | Convex | Entire | 1.5-2 | Rod-shaped | Negative |
| 18 | DO-S3 | Circular | Ivory | Flat | Entire | 1-1.5 | Rod-shaped | Negative |
| 19 | DO-S4 | Circular | Ivory | Convex | Entire | 0.5-1 | Rod-shaped | Negative |


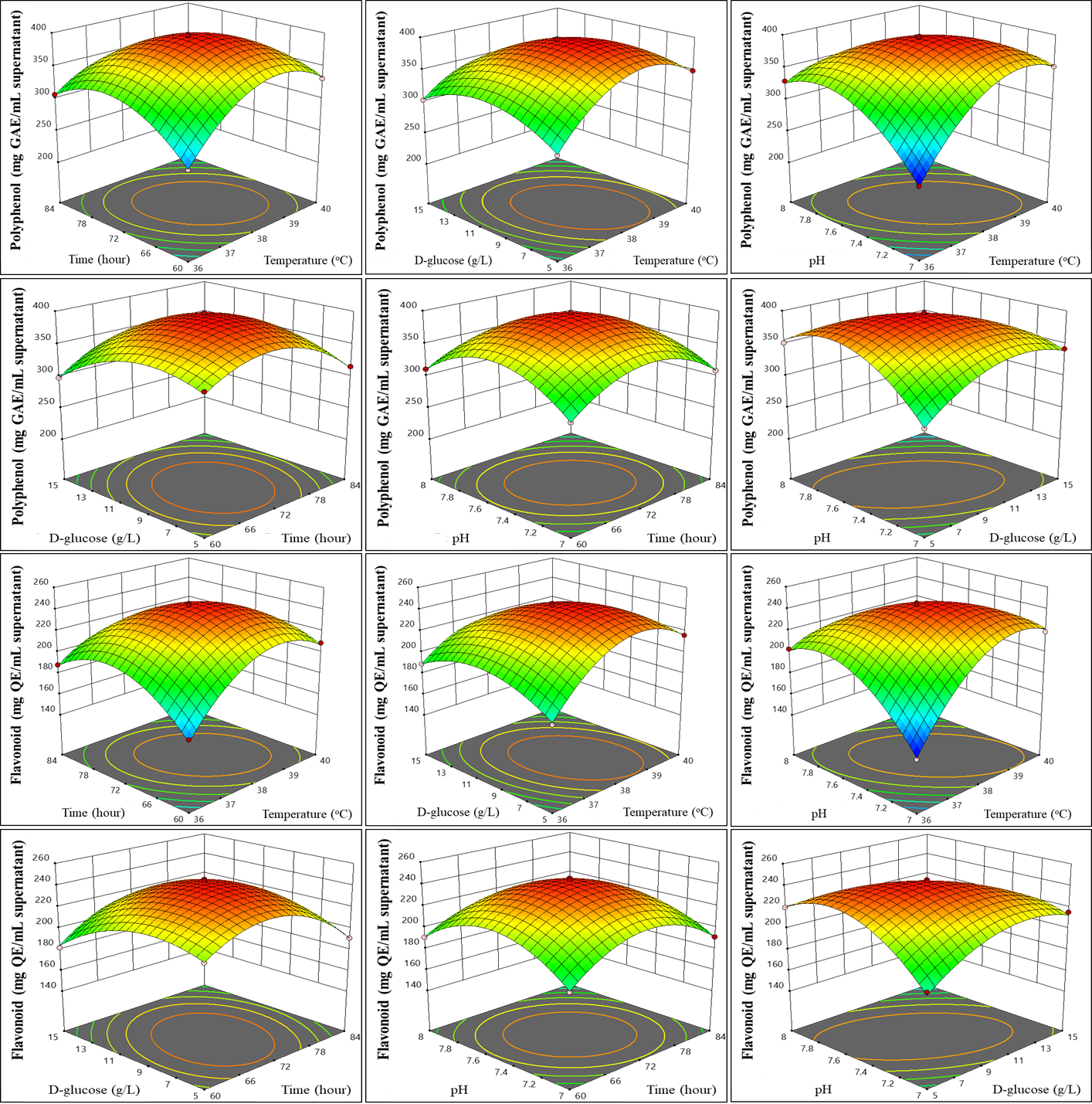


**S. Figure 1.** Response surface of the effects of the interaction of various factors on the ability of bacterial strain DO-R5 to produce polyohenoids and flavonoids

**
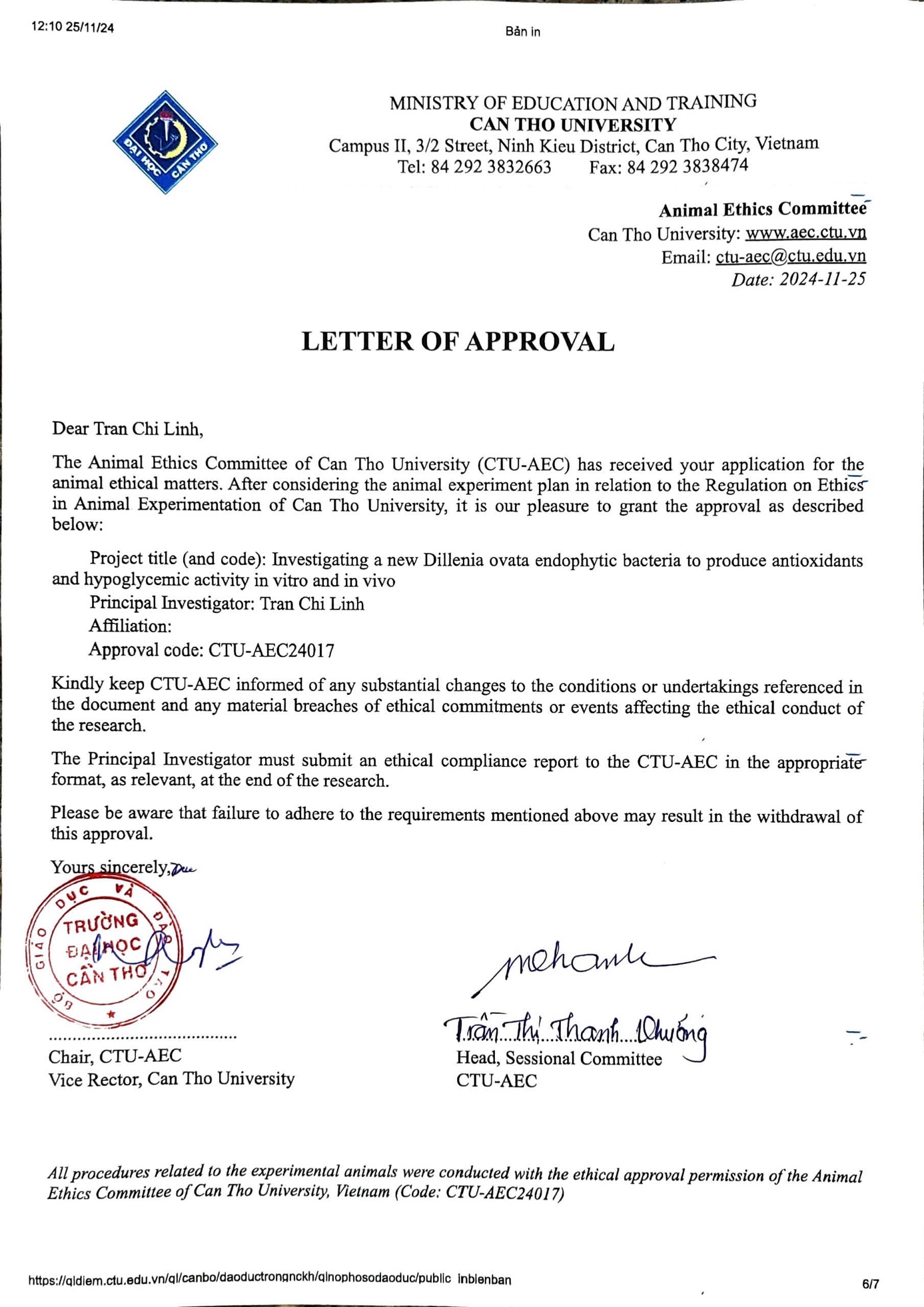
**
